# FcγRIIIA activation-mediated up-regulation of glycolysis alters MDSCs modulation in CD4^+^ T cell subsets of Sjögren syndrome

**DOI:** 10.1038/s41419-023-05631-4

**Published:** 2023-02-06

**Authors:** Jingjing Qi, Xinyang Zhou, Ziran Bai, Zhimin Lu, Xiaolu Zhu, Jiaqing Liu, Junli Wang, Minli Jin, Chang Liu, Xia Li

**Affiliations:** 1 Department of Immunology, College of Basic Medical Science, Dalian Medical University, Dalian, Liaoning 116044 People’s Republic of China; 2 Department of Rheumatology, Affiliated Hospital of Nantong University, Nantong, Jiangsu 226006 People’s Republic of China; 3 Department of Rheumatology and Immunology, Dalian Municipal Central Hospital, Dalian, Liaoning 116083 People’s Republic of China

**Keywords:** Autoimmunity, Mechanisms of disease, Autoimmune diseases

## Abstract

Our and other researchers’ previous studies found that myeloid-derived suppressor cells (MDSCs) were increased, and these MDSCs, supposed to play immunosuppressive roles, showed significant pro-inflammatory effects in Sjögren’s syndrome (SS). However, the key factors and potential mechanisms leading MDSCs to be inflammatory remain unclear. In this study, we found that MDSCs from SS patients were positively correlated with the percentages of Th17 cells, disease activity and serum autoantibodies, and showed higher levels of Fc gamma receptor (FcγR) IIIA and glycolysis. Most importantly, SS MDSCs or heat-aggregated IgG (HAIG)-treated MDSCs down-regulated Th1/Th2 ratio and up-regulated Th17/Treg ratio, which could be obviously rescued by IgG monomer or glycolysis inhibitor 2-DG. As well, the levels of FcγRIV and glycolysis in MDSCs and the ratio of Th17/Treg were increased, and the ratio of Th1/Th2 was decreased in SS-like NOD mice. Our study indicated that MDSCs showed pro-inflammatory phenotypes by disturbing CD4^+^ T-cell balances in SS. The pro-inflammatory effects of MDSCs might be directly linked to the enhanced glycolysis mediated by FcγRIIIA activation.

## Introduction

Sjögren’s syndrome (SS), a chronic systemic autoimmune disease [1], is characterized by lymphocytic infiltration of the exocrine glands, primarily the salivary and lacrimal glands, leading to glandular tissue destruction manifested as ocular (keratoconjunctivitis sicca) and oral (xerostomia) dryness [2, 3]. At time of diagnosis, 75% of SS patients have a high titer of IgG antibodies, which may be cross-reactive against autoantigens to form immune complexes (ICs) [4]. Although life expectancy being unaffected, SS may cause great inconvenience in daily life [5]. Around 5% of patients with primary SS may develop lymphoma [6]. The undefined mechanisms and the lack of effective treatments have hampered the management of patients with SS. Thus, revealing the complex pathophysiological mechanisms and identifying new therapeutic targets are still important challenges for SS therapy.

Myeloid-derived suppressor cells (MDSCs) represent a heterogenous population of immature myeloid cells mainly including monocytic MDSCs (M-MDSCs) and polymorphonuclear MDSCs (PMN-MDSCs), which were firstly identified in tumor infiltration with a capability to support tumor growth and development [7]. Mechanistically, the immunosuppressive function may be related to the activated PI3K-Akt/mTOR pathway in MDSCs of tumor-bearing mice [8]. Interestingly, recent studies have demonstrated that MDSCs were also increased, but played a controversial role in autoimmune diseases. Some studies reported that MDSCs ameliorated systemic lupus erythematosus (SLE) or rheumatoid arthritis (RA) like symptoms in mouse models by inhibiting Th17 cells differentiation or promoting IL-10^+^ B cells expansion [9, 10]. However, other researchers found that MDSCs aggravated SLE or RA in mice by producing IL-1β or B-cell activating factor to promote Th17 cells proliferation or B cells activation [11, 12].

Our previous research has found that MDSCs were increased during the SS-like progression and MDSCs elimination ameliorated SS-like syndrome in mouse model, suggesting a pro-inflammatory role of MDSCs in SS [13]. According to Tian’s reports, MDSCs from early-stage SS mice showed immunosuppressive function on T cells proliferation, but MDSCs from late-stage SS mice almost lost their immunosuppressive capacity and even aggravated the progression of SS in mice [14]. Our and others’ researches further found that the immunosuppressive function of MDSCs was restored in SS mice treated with mesenchymal stem cells [15–17]. All those studies suggest that SS microenvironment impaired the immunosuppressive function and induced the pro-inflammatory effects of MDSCs to promote SS development.

Fc gamma receptors (FcγRs), mainly expressed on myeloid cells, specifically bind to the Fc portion of IgG to regulate cellular effects. Human FcγRs include activating FcγRI, FcγRIIA, FcγRIIC, FcγRIIIA, and FcγRIIIB and inhibitory FcγRIIB. Mouse FcγRs include activating FcγRI, FcγRIII (counterpart of human FcγRIIA) and FcγRIV (counterpart of human FcγRIIIA) and inhibitory FcγRIIB. The multiple antibody Fc fragments present in ICs, but not Fc fragment in monomeric IgG, can induce cellular pro-inflammatory or anti-inflammatory effects by cross-linking with FcγRs to activate immunoreceptor tyrosine-based activation or inhibition motif (ITAM/ITIM) in cytoplasmic region (with the exception of the GPI-anchored FcγRIIIB) [18, 19]. In tumor microenvironment, the activation of FcγRIIIA or FcγRIIB could suppress or enhance MDSCs immunosuppressive capacity on T cells proliferation [20, 21]. Given that the elevated serum autoantibodies, which can form immune complexes by binding to autoantigens, are crucial clinical characteristics of SS, we supposed that IgG-containing ICs may be involved in inducing MDSCs inflammation effects by binding to FcγRs.

According to the published reports, the metabolites of glycolysis are important for MDSCs development and activation in cancer [22]. Our previous study found that the activation of mammalian target of rapamycin (mTOR), a key kinase in cellular metabolisms, could mediate MDSCs to induce the unbalance of Th17/Treg in lupus mice [23]. Thus, we sought to investigate the effects of FcγRs activation on MDSCs and whether the cellular metabolisms reprogramming might provide insight into molecular mechanisms for these events. We found that SS-MDSCs were positively correlated with the percentages of Th17 cells, disease activity and serum autoantibodies and showed high levels of FcγRIIIA and glycolysis. Mechanistically, the altered MDSCs modulation in CD4^+^ T cells might be directly linked to the enhanced glycolysis induced by FcγRIIIA activation in SS.

## Material and methods

### Patients and healthy controls

All patients (*n* = 50) were diagnosed with SS according to the American-European Consensus Group criteria for primary SS or the 2012 American College of Rheumatology criteria in The Second Hospital of Dalian Medical University. Detailed clinical characteristics and laboratory features were shown in Table S1. Informed consent was obtained from all patients according to the principles of the Declaration of Helsinki. Age/sex-matched healthy controls (HC, *n* = 60) were served as controls. All participants were given written informed consent, and all procedures in this study were approved by the ethics committee of The Second Hospital of Dalian Medical University.

### Mice

5 and 13-week-old female non-obese diabetic (NOD) mice (*n* = 5 per group) were obtained from the Model Animal Research Center of Nanjing University and kept under pathogen-free conditions in the animal center of Dalian Medical University Medical School.

### Flow cytometry

For MDSCs quantification, cells were stained in PBS with anti-human HLA-DR-PE/Cy7 (eBioscience), anti-human CD11b-PerCP/Cy5.5 (Biolegend) and anti-human CD33-Alexa Fluor 647 (Biolegend) or anti-mouse CD11b-PreCP/Cy5.5 (Biolegend) and anti-mouse Gr-1-PE/Cy7 (Biolegend).

For FcγRs, glucose uptake capacity and glycolysis relevant molecules quantification, cells were stained in PBS with anti-human FcγRIII (CD16)-Alexa Fluor 488 (Biolegend), anti-human FcγRII (CD32)-PE/Cy5.5 (eBioscience), 2-NBDG (2-deoxyglucose analogue), anti-Glut1-PE (BD Biosciences), anti-HK2-Alexa Fluor 647 (abcam), anti-LDH-PE (abcam), anti-p-mTOR-PE (eBioscience), anti-HIF-1α-PE (Biolegend), or anti-mouse FcγRIV (CD16-2)-FITC (Biolegend).

For Th1, Th2, and Th17 cells quantification, cells were pelleted by centrifugation and resuspended in 10% FBS RPMI-1640 medium with phorbol 12-myristate 13-acetate (PMA, 200 ng/ul) and ionomycin (1 μg/ml) and brefeldin A (5 μg/ml, eBioscience) at 37 °C for 4 h. Cells were washed twice in cold PBS and stained with anti-human CD4-PerCP/Cy5.5 (eBioscience) or anti-mouse CD4-PE/Cy7 (Biolegend). After permeabilization with Cytofix/Cytoperm (BD Biosciences, San Diego, USA), cells were stained with anti-human IFN-γ-APC (eBioscience), anti-human IL-4-PE (eBioscience), anti-human IL-17A-PE (eBioscience) or anti-mouse IFN-γ-APC (eBioscience), anti-mouse IL-4-PE (eBioscience), anti-mouse IL-17A-PE (eBioscience).

For Treg cells quantification, cells were washed twice in cold PBS and stained with anti-human CD4-PerCP/Cy5.5 (Biolegend) or anti-mouse CD4-PE/Cy7 (Biolegend). After fixation and permeabilization by Transcription Factor Staining Buffer Set (eBioscience) overnight at 4 °C, cells were stained with anti-human Foxp3-PE (eBioscience) or anti-mouse Foxp3-PE (eBioscience).

### Lactate assay

HC and SS serum samples were diluted properly for the measurement of lactate level with a Lactate Assay Kit II (Abbkine Scientific, China).

### Generation of human MDSCs

CD14^+^ monocytes were isolated from peripheral blood mononuclear cell (PBMC) of HC and SS patients by microbeads (Miltenyi Biotec) according to the manufacturer’s instructions. The cells were stimulated with human GM-CSF (80 ng/ml) and human IL-6 (80 ng/ml) in 10% FBS RPMI-1640 medium at 37 °C with 5% CO_2_ for 15 days. The medium was changed every 5 days.

### Heat-aggregated IgG preparation

Serum samples of SS patients with positive anti-SSA antibodies were collected, and IgG was isolated using HiTrap Protein G according to the manufacturer’s instructions. IgG aggregates were obtained by heat aggregation at 63 °C for 12 min. The heat-aggregated IgG (HAIG) was centrifuged at 10,000*g* for 5 min and the precipitate was discarded. The soluble HAIG in supernatant was diluted with PBS to 1 mg/ml for storage. HC-MDSCs were stimulated with IgG or HAIG at the final concentration of 100 μg/ml.

### Co-culture of MDSCs and CD4^**+**^ T cells

Some MDSCs were preprocessed with 100 μg/ml IgG or 1 mM 2-deoxy-d-glucose (2-DG, Solarbio, China) for 1 h. Then, MDSCs or preprocessed MDSCs were stimulated with IgG or HAIG for 24 h. HC CD4^+^ T cells labeled with CFSE (2.5 μM) were cultured alone or with MDSCs (2:1) in the presence of human anti-CD3/CD28 antibody (2 μg/ml each) for 3 days. CD4^+^CFSE^low^ T cells were measured by flow cytometer.

### Quantitative real-time PCR

Cells were collected and total RNA were prepared using TRIzol reagent (AG, AG21102). Genes expression was quantified relative to that of GAPDH using the thermal cycler (Bio-Rad, USA) and normalized by standard 2^−ΔΔCT^ calculation. Primer sequences were listed in Table S2.

### Statistical analysis

Data is presented as means ± standard errors of the mean (SEM). GraphPad Prism was used to conduct all statistical analyses. The parametric Student’s *t*-test was used for comparison of two groups. One-way ANOVA was used for multiple comparisons. Correlations were determined by Spearman chi-square tests. In all analyses, *p* < 0.05 was considered statistically significant.

## Results

### Increased peripheral MDSCs of SS patients were positively correlated with clinical index

**Fig. 1:**
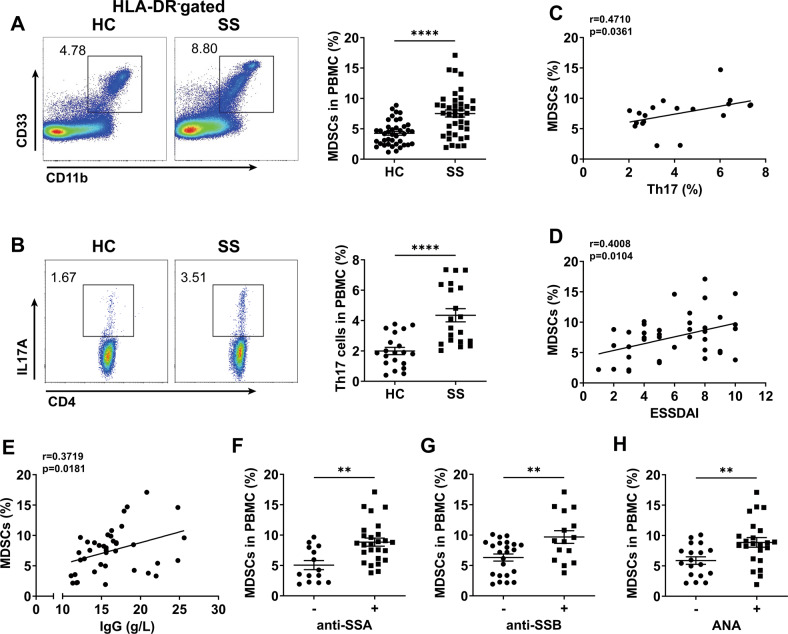
Peripheral MDSCs from SS patients were positively correlated with the percentages of Th17 cells and disease activity. **A**, **B** Human peripheral HLA-DR^-^CD11b^+^CD33^+^ MDSCs and CD4^+^IL-17A^+^ Th17 cells (HC *n* = 40, SS *n* = 40) were detected by flow cytometry. Representative flow cytometric analysis and percentages of MDSCs in HC and SS patients were shown. **C**–**E** The relationships of MDSCs with Th17 cells, EULAR Sjögren’s Syndrome Disease Activity Index (ESSDAI) and serum IgG were analyzed (*n* = 40). **F**–**H** SS patients were divided into antibodies-positive (+) and antibodies-negative (–) two groups, whose percentages of MDSCs were compared. **p < 0.01, ****p < 0.0001.

Our previous researches have found that MDSCs and Th17 cells were increased significantly in SS patients [13, 24]. Here, we added some new samples to determine the correlations of peripheral MDSCs with Th17 cells and clinical index in SS patients. The results confirmed that the percentages of MDSCs and Th17 cells were both higher in SS patients than those in HC (Fig. 1A, B). Further, MDSCs were positively correlated with Th17 cell percentages (Fig. 1C), disease activity (ESSDAI) (Fig. 1D) and serum IgG levels (Fig. 1E) in SS patients. In addition, compared to those SS patients with anti-SSA or anti-SSB or antinuclear antibodies (ANA) negative, SS patients with these specific autoantibodies positive showed higher levels of MDSCs (Fig. 1F–H). These data suggest that MDSCs may be involved in the disease progression of SS patients.

### Peripheral MDSCs of SS patients induced the imbalance of Th1/Th2 and Th17/Treg cell subsets

**Fig. 2:**
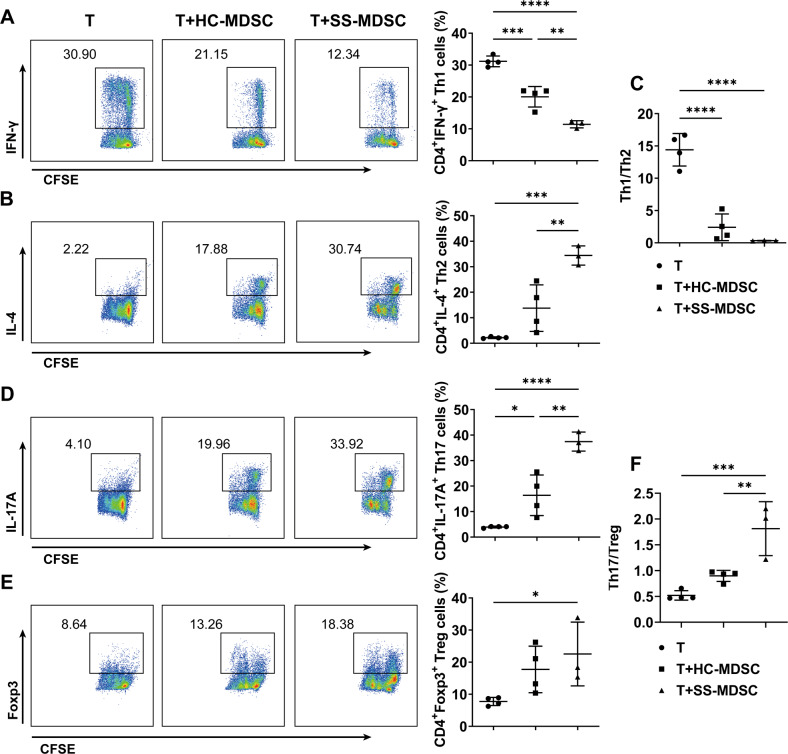
MDSCs disturbed the balances of Th1/Th2 and Th17/Treg cell subsets in SS patients. Peripheral monocytes from HC and SS patients were induced into MDSCs, which were co-cultured with CFSE-labeled CD4^+^ T cells for 72 h. Representative flow cytometric analysis and percentages of CD4^+^ T cell subsets were shown. **A** CD4^+^IFN-γ^+^ Th1 cells, **B** CD4^+^IL-4^+^ Th2 cells, **C** the ratio of Th1/Th2, **D** CD4^+^IL-17A^+^ Th17 cells, **E** CD4^+^Foxp3^+^ Treg cells and **F** the ratio of Th17/Treg. All the data are representative of two independent experiments. *n* = 4, *p < 0.05, **p < 0.01, ***p < 0.001, ****p < 0.0001.

Studies have found that the dysfunctional MDSCs could promote the progression of SS mice by enhancing Th1 and Th17 responses [14]. In the present study, we will further verify the possible pathogenic effects of MDSCs from SS patients on CD4^+^ T cell subsets which are involved in the pathogenesis of SS. MDSCs were induced from HC or SS patients’ peripheral monocytes and co-cultured with HC CD4^+^ T cells. Flow cytometric analysis showed that both HC-MDSCs and SS-MDSCs inhibited CD4^+^ T cells proliferation (Fig. S1), which is the primary characteristic of MDSCs. More importantly, we found that compared to HC-MDSCs, SS-MDSCs obviously decreased the proportion of Th1 cells (Fig. 2A), but increased the proportion of Th2 cells (Fig. 2B), Th17 cells (Fig. 2D) and Treg cells (Fig. 2E). However, SS-MDSCs significantly downregulated the ratio of Th1/Th2 (Fig. 2C) and upregulated the ratio of Th17/Treg (Fig. 2F). These results suggest that MDSCs of SS patients might play an inflammatory role by disturbing the balances of Th1/Th2 and Th17/Treg cell subsets.

### FcγRIIIA activation altered SS-MDSCs modulation in CD4^+^ T cell subsets

**Fig. 3:**
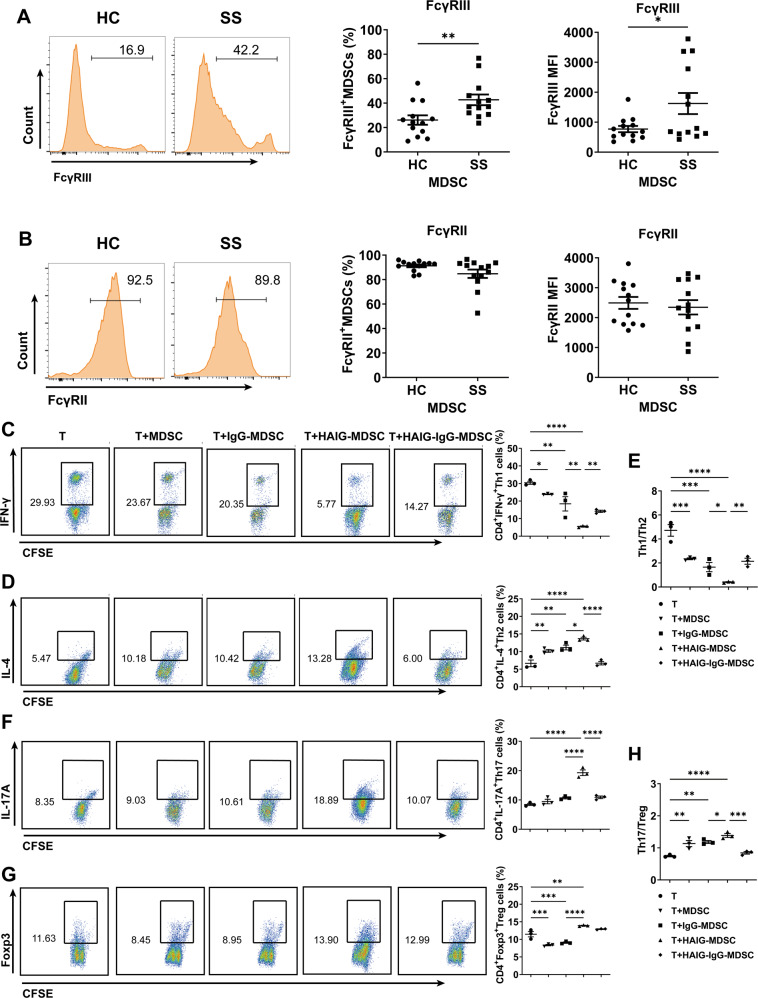
FcγRIIIA activated MDSCs disturbed the balances of Th1/Th2 and Th17/Treg cell subsets. **A**, **B** FcγRIII and FcγRII on peripheral MDSCs (HC *n* = 13, SS *n* = 13) were detected by flow cytometry. Representative flow cytometric analysis and percentages of FcγRIII^+^ MDSCs and FcγRII^+^ MDSCs and the MFI of FcγRIII and FcγRII on MDSCs from HC and SS patients were shown. **C**–**H** MDSCs or IgG-preprocessed MDSCs were stimulated with or without IgG or HAIG for 24 h and then co-cultured with CFSE-labeled CD4^+^ T cells for 72 h. Representative flow cytometric analysis and percentages of CD4^+^ T cell subsets were shown. **C** CD4^+^IFN-γ^+^ Th1 cells, **D** CD4^+^IL-4^+^ Th2 cells, **E** the ratio of Th1/Th2, **F** CD4^+^IL-17A^+^ Th17 cells, **G** CD4^+^Foxp3^+^ Treg cells, **H** the ratio of Th17/Treg. All the data are representative of two independent experiments. *n* = 4, *p < 0.05, **p < 0.01, ***p < 0.001, ****p < 0.0001.

Given that the elevated serum autoantibodies are crucial clinical characteristics of SS, and there were positive correlations of MDSCs with the levels of serum IgG, anti-SSA antibodies, anti-SSB antibodies, and ANA, we wonder whether IgG-containing SS medium can result in SS-MDSCs dysfunction to disturb CD4^+^ T-cell balances. Thus, we detected and compared the levels of FcγRII and FcγRIII on peripheral MDSCs from HC and SS patients. Flow cytometric analysis showed that the percentages of FcγRIII^+^ MDSCs and the mean fluorescence intensity (MFI) of FcγRIII on MDSCs were significantly higher in SS patients than those in HC (Fig. 3A). The percentages of FcγRII^+^ MDSCs and the MFI of FcγRII on MDSCs were comparable in HC and SS patients (Fig. 3B). Further, we found that M-MDSCs, but not PMN-MDSCs were positively correlated with serum IgG levels (Fig. S2B and C) and expressed higher level of FcγRIIIA in SS patients. These data indicate that the pro-inflammatory effects of MDSCs of SS patients might be due to the aberrant expression and activation of FcγRIIIA.

Next, we will confirm whether the pro-inflammatory effects of MDSCs are induced by FcγRIIIA activation. According to the published reports, only the cross-linking of multiple antibody Fc fragments present in immune complexes, but not monomeric IgG, with FcrRs can induce effector cells activation [19]. We heated the purified serum IgG into heat-aggregated IgG (HAIG) which simulates ICs in SS medium, and then MDSCs stimulated with or without IgG or HAIG were co-cultured with CFSE-labeled CD4^+^ T cells for 72 h. As we expected, HAIG-MDSCs significantly inhibited CD4^+^ T cells proliferation (Fig. S4A) and decreased the proportion of Th1 cells (Fig. 3C) but increased the proportion of Th2 cells (Fig. 3D), Th17 cells (Fig. 3F) and Treg cells (Fig. 3G), and decreased the ratio of Th1/Th2 (Fig. 3E) and increased the ratio of Th17/Treg (Fig. 3H), compared to MDSCs and IgG-MDSCs. What’s more, all HAIG-induced effects of MDSCs could be rescued by IgG monomer. And qPCR results showed that HAIG stimulation increased the expression of FcγRIIIA, but not FcγRI, FcγRIIA, and FcγRIIB in MDSCs (Fig. S3A). These results demonstrate that IgG^+^ ICs-mediated FcγRIIIA activation disturbed SS-MDSCs modulation in the balances of Th1/Th2 and Th17/Treg cell subsets.

### MDSCs of SS patients showed enhanced glycolysis by FcγRIIIA activation, which contributed to the aberrant modulation in CD4^+^ T cells of SS-MDSCs

**Fig. 4:**
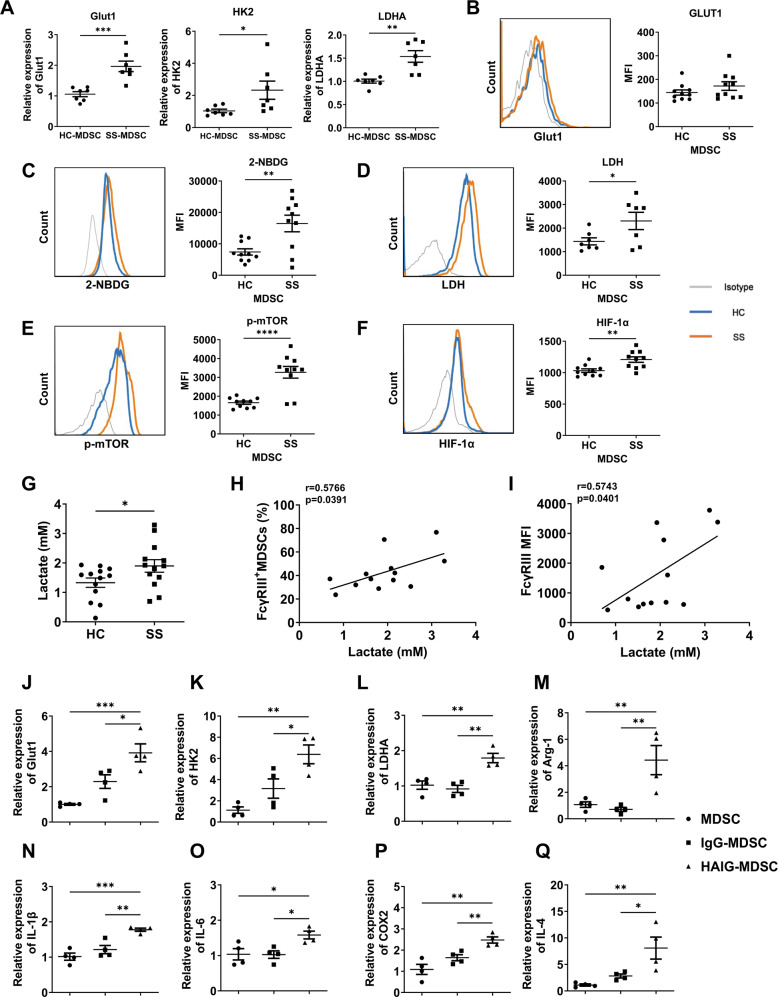
Peripheral MDSCs of SS patients showed enhanced glycolysis levels, which was promoted by FcγRIIIA activation. **A** Peripheral monocytes from HC and SS patients were induced into MDSCs. The mRNA expressions of Glut1, HK2, and LDHA in HC and SS-MDSCs were detected by qPCR (*n* = 7). **B**–**F** Peripheral HLA-DR^-^CD11b^+^CD33^+^ MDSCs were gated to analyze the glucose uptake capacity and protein levels of key factors in glycolytic pathway. Representative flow cytometric analysis and MFI of **B** Glut1, **C** 2-NBDG, **D** LDH, **E** p-mTOR and **F** HIF-1α in MDSCs were shown (*n* = 10 or 7). **G** The levels of lactate in HC and SS serum were quantified with the Lactate Assay Kit II, **H**–**I** and the correlations of lactate levels with FcγRIII^+^ MDSCs and the FcγRIII MFI of MDSCs in SS patients were analyzed (*n* = 13). **J**–**Q** Peripheral monocytes from HC and SS patients were induced into MDSCs, which were stimulated with IgG and HAIG for 24 h (*n* = 4). The mRNA expressions of **J** Glut1, **K** HK2, **L** LDHA, **M** Arg-1, **N** IL-1β, **O** IL-6, **P** COX2, and **Q** IL-4 were detected by qPCR. All the data are representative of two independent experiments. *p < 0.05, **p < 0.01, ***p < 0.001, ****p < 0.0001.

Glycolytic metabolites are thought to be important factors in regulating cell fate and inflammation. Our previous studies found that the activation of mTOR, a key kinase in cellular metabolisms, could mediate MDSCs to induce the unbalance of Th17/Treg in lupus mice [25]. Thus, we sought to determine whether FcγRIIIA activation can induce abnormal glycolysis which contributes to the MDSCs dysfunction of SS patients. Firstly, we detected the mRNA expressions of glycolysis-associated indexes glucose transporter 1 (Glut1), hexokinase 2 (HK2) and lactate dehydrogenase A (LDHA) in HC and SS-MDSCs by qPCR. The results showed that SS-MDSCs showed higher mRNA expressions of Glut1, HK2, and LDHA than HC-MDSCs (Fig. 4A). And the analysis with flow cytometry confirmed the higher levels of glucose uptake capacity (Fig. 4C), LDH expression (Fig. 4D), mTOR phosphorylation (Fig. 4E) and transcription factor HIF-1α expression (Fig. 4F) in SS-MDSCs than those in HC-MDSCs. In addition, serum lactate levels were higher in SS patients than those in HC (Fig. 4G), which were positively correlated with FcγRIII^+^ MDSCs (Fig. 4H) and the FcγRIII MFI of MDSCs (Fig. 4I) in SS patients.

Next, we would investigate whether enhanced glycolysis of SS-MDSCs was associated with FcγRIIIA activation. SS-MDSCs were stimulated with or without IgG monomer or HAIG to determine the expressions of glycolysis relevant molecules and functional factors. QPCR results showed that HAIG-stimulated MDSCs had increased mRNA expressions of Glut1, HK2, and LDHA (Fig. 4J–L), as well as Arg-1, IL-1β, IL-6, COX2, and IL-4 (Fig. 4M–Q). These results suggest that the MDSCs metabolism was reprogrammed to enhanced glycolysis in SS patients.

**Fig. 5:**
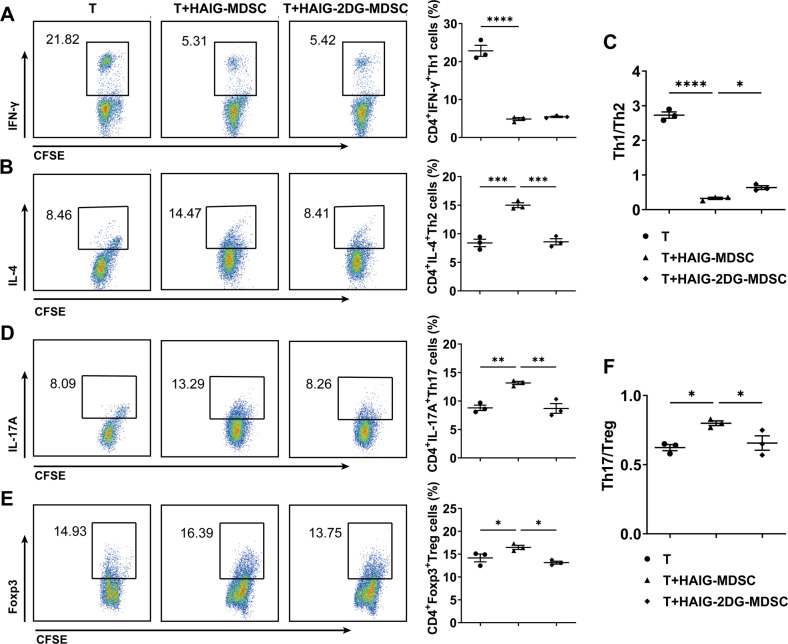
Glycolysis inhibition rescued MDSC dysfunction induced by HAIG. **A**–**F** MDSCs or 2-DG-preprocessed MDSCs were stimulated with or without HAIG for 24 h and then co-cultured with CFSE labeled CD4^+^ T cells for 72 h. Representative flow cytometric analysis and percentages of CD4^+^ T cell subsets were shown. **A** CD4^+^IFN-γ^+^ Th1 cells, **B** CD4^+^IL-4^+^ Th2 cells, **C** the ratio of Th1/Th2, **D** CD4^+^IL-17A^+^ Th17 cells, **E** CD4^+^Foxp3^+^ Treg cells and **F** the ratio of Th17/Treg. All the data are representative of two independent experiments. *n* = 3, *p < 0.05, **p < 0.01, ***p < 0.001, ****p < 0.0001.

To further explore the role of glycolysis in FcγRIIIA activation-induced MDSC dysfunction, MDSCs stimulated with or without HAIG or glycolysis inhibitor 2-DG were co-cultured with CFSE labeled CD4^+^ T cells for 72 h. Flow cytometric analysis showed that glycolysis inhibition had no significant effects on HAIG-MDSCs modulation in CD4^+^T cells proliferation (Fig. S5A) and Th1 cells expansion (Fig. 5A), but rescued the effects of HAIG-MDSCs on Th2, Th17, Treg cells expansion and the alteration of Th1/Th2 and Th17/Treg balances (Fig. 5B–F). These results suggest that enhanced glycolysis by FcγRIIIA activation contributed to the aberrant modulation of SS-MDSCs in CD4^+^ T cells.

### SS-like NOD mice showed higher levels of FcγRIV and glycolysis in MDSCs and disturbed CD4^+^ T-cell balances

**Fig. 6:**
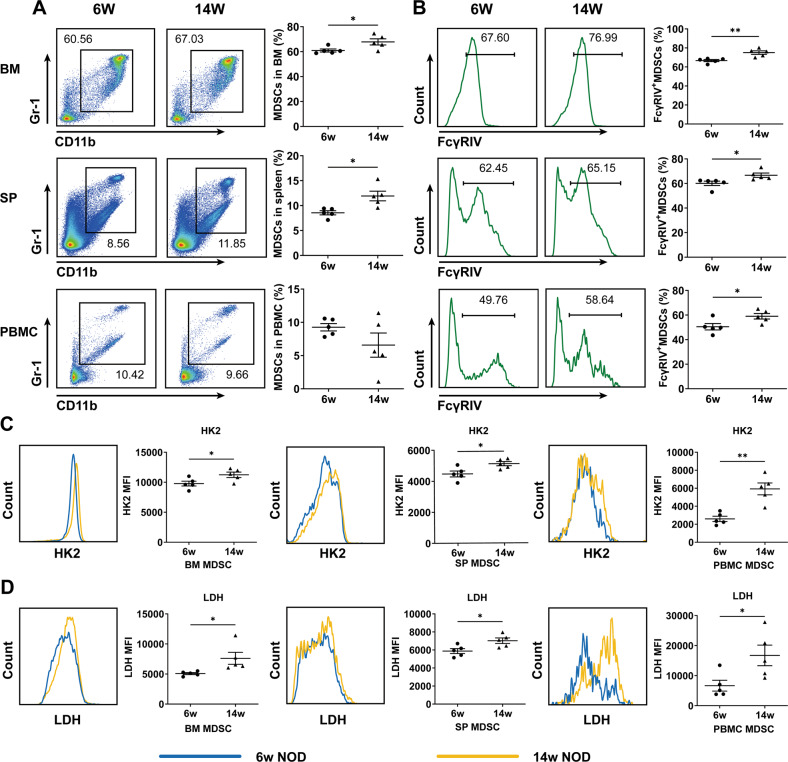
MDSCs highly expressed FcγRIV and glycolysis-associated factors in SS-like NOD mice. CD11b^+^Gr-1^+^ MDSCs in BM, SP, and PBMC from 6 and 14-week-old NOD mice were detected by flow cytometry. Representative flow cytometric analysis and percentages of **A** MDSCs and **B** FcγRIV^+^ MDSCs in BM, SP, and PBMC (from the top down) were shown. Representative flow cytometric analysis and MFI of **C** HK2 and **D** LDH in MDSCs were shown. All the data are representative of two independent experiments. *n* = 5, *p < 0.05, **p < 0.01.

Our previous study found that NOD mice showed significant SS-like symptoms at 8th week and MDSCs were increased with the development of SS-like syndrome [13]. In the present study, we will clarify the potential mechanism for the pathogenic roles of MDSCs in NOD mice with SS-like symptoms. Firstly, we showed that compared to 6-week-old NOD mice (without SS-like symptoms), 14-week-old NOD mice (with SS-like symptoms) showed higher levels of MDSCs in bone marrow (BM), spleen (SP) and lymph node (LN), but not PBMC (Fig. 6A and Fig. S6A). Additionally, MDSCs from BM, SP, PBMC and LN highly expressed FcγRIV (the mouse counterpart of human FcγRIIIA) (Fig. 6B and Fig. S6B) and glycolysis-associated HK2 and LDH (Fig. 6C, D) in 14-week-old NOD mice.

**Fig. 7:**
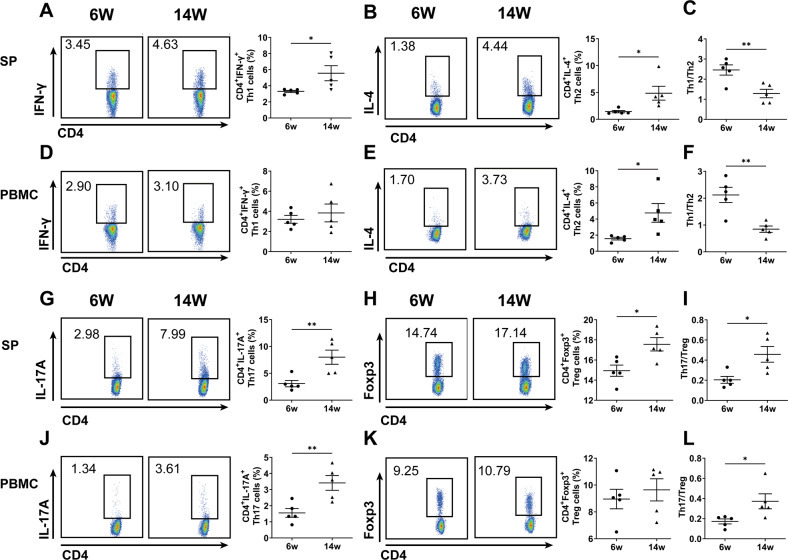
Th1/Th2 and Th17/Treg were imbalanced in SS-like NOD mice. CD4^+^IFN-γ^+^ Th1 cells, CD4^+^IL-4^+^ Th2 cells, CD4^+^IL-17A^+^ Th17 cells and CD4^+^Foxp3^+^ Treg cells in SP and PBMC from 6 and 14-week-old NOD mice were detected by flow cytometry. Representative flow cytometric analysis and percentages of CD4+ T cell subsets were shown. **A** Th1 cells in SP, **B** Th2 cells in SP, **C** the ratio of Th1/Th2 in SP, **D** Th1 cells in PBMC, **E** Th2 cells in PBMC, **F** the ratio of Th1/Th2 in PBMC, **G** Th17 cells in SP, **H** Treg cells in SP, **I** the ratio of Th17/Treg in SP, **J** Th17 cells in PBMC, **K** Treg cells in PBMC, **L** the ratio of Th17/Treg in PBMC. All the data are representative of two independent experiments. *n* = 5, *p < 0.05, **p < 0.01.

What’s more, the percentages of Th1, Th2, Th17, and Treg cells were increased in SP, PBMC or LN (Fig. 7A–H and Fig. S6C–D and F–G), and the ratio of Th1/Th2 was decreased significantly in SP (Fig. 7C) and PBMC (Fig. 7F), and the ratio of Th17/Treg was increased significantly in SP (Fig. 7I), PBMC (Fig. 7L) and LN (Fig. S6H) from 14-week-old NOD mice. All these results suggest that FcγRIV and glycolysis levels of MDSCs were increased and the balances of Th1/Th2 and Th17/Treg cell subsets were disturbed in SS-like NOD mice.

## Discussion

**Fig. 8:**
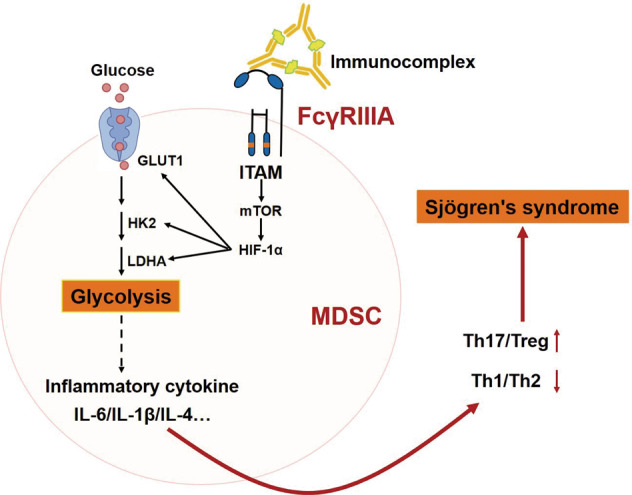
Schematic representation of FcγRIIIA activationinducing MDSCs to alter CD4^+^ T cell subsets by enhancing glycolysis in Sjögren syndrome. By cross-linking with FcγRIIIA on MDSCs, immunocomplex activates the cytoplasmic ITAM, which induces the activation of mTOR to promote the expression of transcriptional factor HIF-1α. Furthermore, HIF-1α promote the expression of glycolysis-associated Glut1, HK2 and LDHA to enhance glycolysis, which results in inflammatory cytokines up-regulation leading to the decreased Th1/Th2 ratio and increased Th17/Treg ratio.

Despite MDSCs were defined by their immunosuppressive nature, their complex pathological characterizations still have not been elucidated clearly in autoimmune diseases. Our previous studies found that the increased MDSCs showed significant pro-inflammatory effects in SS [7, 13, 17]. In the present study, we further clarify that SS-MDSCs highly express FcγRIIIA and glycolysis-associated factors. More importantly, we confirm that the up-regulated glycolysis in SS-MDSCs by FcγRIIIA activation contributes to imbalances of Th1/Th2 and Th17/Treg cell subsets. Meanwhile, we elucidate that the levels of FcγRIV and glycolysis in MDSCs and the ratio of Th17/Treg are increased, and the ratio of Th1/Th2 is decreased in SS-like NOD mice (Fig. 8).

Because of the immunosuppressive activities, MDSCs have been taken as a promising target to improve cancer immune therapy [26]. However, MDSCs have also been observed increasing in diverse inflammatory settings, and this MDSC concept has hampered studies on their identification, experimental manipulation and direct contribution in various pathological contexts [27]. Tian and colleagues found that MDSCs from early-stage SS mice could attenuate SS-like symptoms by inhibiting Th1 and Th17 cell responses significantly, but, MDSCs from late-stage SS mice aggravated SS-like symptoms by promoting Th1 and Th17 cell responses in mice [14]. Our present study found that compared to HC-MDSCs, SS-MDSCs decreased the ratio of Th1/Th2, but increased the ratio of Th17/Treg significantly. These conflicting data of MDSCs function in different studies may due to the multiple roles of these cells in different differentiation stages and disease contexts.

FcγRs are involved in promoting or suppressing autoimmune diseases and FcγR-targeting molecules can be effective therapeutics to improve symptom severity [28, 29]. FcγRI is high-affininty receptor with the capacity of binding to IgG monomer and IgG-containing ICs. FcγRII and FcγRIII are low-affininty receptors mainly binding to IgG-containing ICs. FcγRI, FcγRIIA, FcγRIIB, and FcγRIIIA are widely expressed on myeloid cells and lymphocytes. FcγRIIC is expressed by a minority of individuals depending on the allelic status and FcγRIIIB is only expressed by neutrophils [30]. Following cross-linking with immune complexes, ITAM signaling pathways initiated by different types of activating FcγRs are quite similar [19]. In this study, we showed that IgG-containing ICs induced MDSCs dysfunction mainly via activating FcγRIIIA in SS patients. However, the levels of FcγRIIA, FcγRIIC and FcγRIIB were not detected respectively due to lacking of the specific antibodies. The exact roles of FcγRIIA, FcγRIIC, and FcγRIIB need further investigation.

According to the published reports, tumor glycolysis and the glycolytic metabolites in MDSCs are crucial for MDSCs survival, accumulation and immunosuppressive functions [22, 31, 32]. But the underlying molecular mechanisms associated with glycolysis to modulate MDSCs fate and function remain unknown. Emerging evidence reveals that a lot of metabolites have immunomodulatory functions by regulating gene transcription and post-translational modification or playing cytokine-like roles [33–35]. In the present study, we found that FcγRIIIA activation increased the expression of LDHA in MDSCs. LDH has been shown to repress translation of cytokines by binding the AU-rich element within 3′ UTRs of mRNA. However, the activation signals in T cells force LDH to releases these mRNAs to catalyze the interconversion of pyruvate and lactate, allowing the translation of these inflammatory cytokines [36]. However, the function of LDHA as the RNA-binding protein in MDSCs still needs further investigation. In addition, the increased lactate may activate MDSCs to produce higher level of pro-inflammatory mediators or promote T cells differentiate into Th17 cells to aggravate inflammation.

Based on the previous results, our current work further explored the key factors and mechanisms inducing MDSCs dysfunction, and obtained the preliminary conclusion that the pro-inflammatory effects of MDSCs might be directly linked to the enhanced glycolysis induced by FcγRIIIA activation in SS. Whereas, more direct evidences of FcγRIIIA activation inducing MDSCs pro-inflammatory effects by enhancing glycolytic activity in SS patients are still needed to be explored.

### Clinical perspectives

MDSCs are increased and play a significant pro-inflammatory role in Sjögren’s syndrome (SS). However, the key factors and mechanisms leading MDSCs to be inflammatory remain unclear.

MDSCs are positively correlated with Th17 cells, disease activity, and serum autoantibodies, and show higher levels of FcγRIIIA and glycolysis in SS patients. FcγRIIIA activation-mediated glycolysis alters MDSCs modulation in the balances of Th1/Th2 and Th17/Treg cell subsets.

Our results highlight a role for FcγRIIIA in MDSCs inflammatory effects and targeting FcγRIIIA activated MDSCs can serve as a novel target for SS and other autoimmune diseases treatment.

## Supplementary information

FcγRIIIA activation-mediated up-regulation of glycolysis alters MDSCs modulation in CD4+ T cell subsets of Sjögren Syndrome

## Data Availability

All data relevant to the present study are included in the article or uploaded as online supplementary information. All data are available upon request.
