# Supplementary material for: FcγRIIIA activation-mediated up-regulation of glycolysis alters MDSCs modulation in CD4+ T cell subsets of Sjögren syndrome
Source: Cell Death Dis. 2023 Feb 6;14(2):86. doi: 10.1038/s41419-023-05631-4 (PMC9902521; doi:10.1038/s41419-023-05631-4)
Supplement: Supplementary file 1 — FcγRIIIA activation-mediated up-regulation of glycolysis alters MDSCs modulation in CD4+ T cell subsets of Sjögren Syndrome [file 41419_2023_5631_MOESM1_ESM.docx]

**Research Article**

**FcγRIIIA activation-mediated up-regulation of glycolysis alters MDSCs modulation in CD4^+^ T cell subsets of Sjögren Syndrome**

Jingjing Qi^1^*, Xinyang Zhou^1^*, Ziran Bai^1^, Zhimin Lu^1, 2^, Xiaolu Zhu^1^, Jiaqing Liu^1^, Junli Wang^1^, Minli Jin^1^, Chang Liu^3🖂^, Xia Li^1🖂^

^1^Department of Immunology, College of Basic Medical Science, Dalian Medical University, Dalian, Liaoning, 116044, People’s Republic of China. ^2^Department of Rheumatology, Affiliated Hospital of Nantong University, Nantong, Jiangsu, 226006, People’s Republic of China. ^3^Department of Rheumatology and Immunology, Dalian Municipal Central Hospital, Dalian, Liaoning, 116083, People’s Republic of China.

**Correspondence:** Xia Li (lixia0416@dmu.edu.cn) and Chang Liu (drlc0820@163.com)

^🖂^Chang Liu and Xia Li share senior authorship.

*Jingjing Qi and Xinyang Zhou contributed equally to this work.

**Running title:** FcγRIIIA activated MDSCs alter CD4^+^ T cells in SS

**Conflict of interest:** none

**Supplementary data**

**
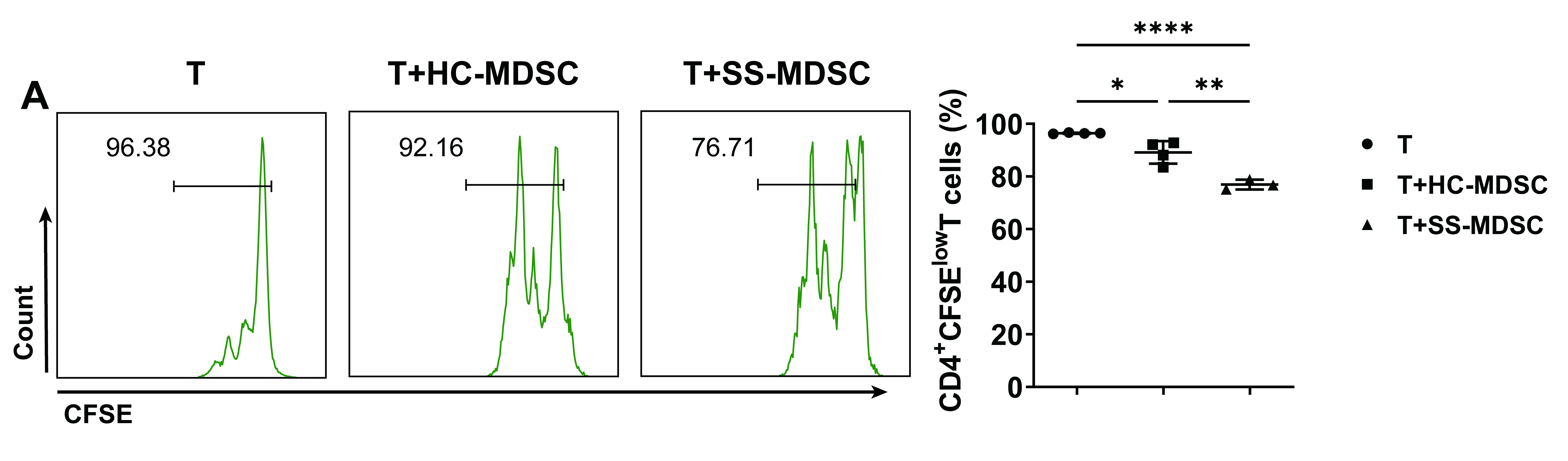
**

**Fig. S1. Enhanced** **suppressive ability of MDSCs** **in CD4^+^ T cell proliferation in SS patients.** Peripheral monocytes from HC and SS patients were induced into MDSCs, and then were co-cultured with CFSE labeled CD4^+^ T for 72 h. **(A)** Representative flow cytometric analysis and percentages of CD4^+^CFSE^low^ T cells were showed. All the data are representative of two independent experiments. n=4, *p<0.05, **p<0.01, ****p<0.0001.


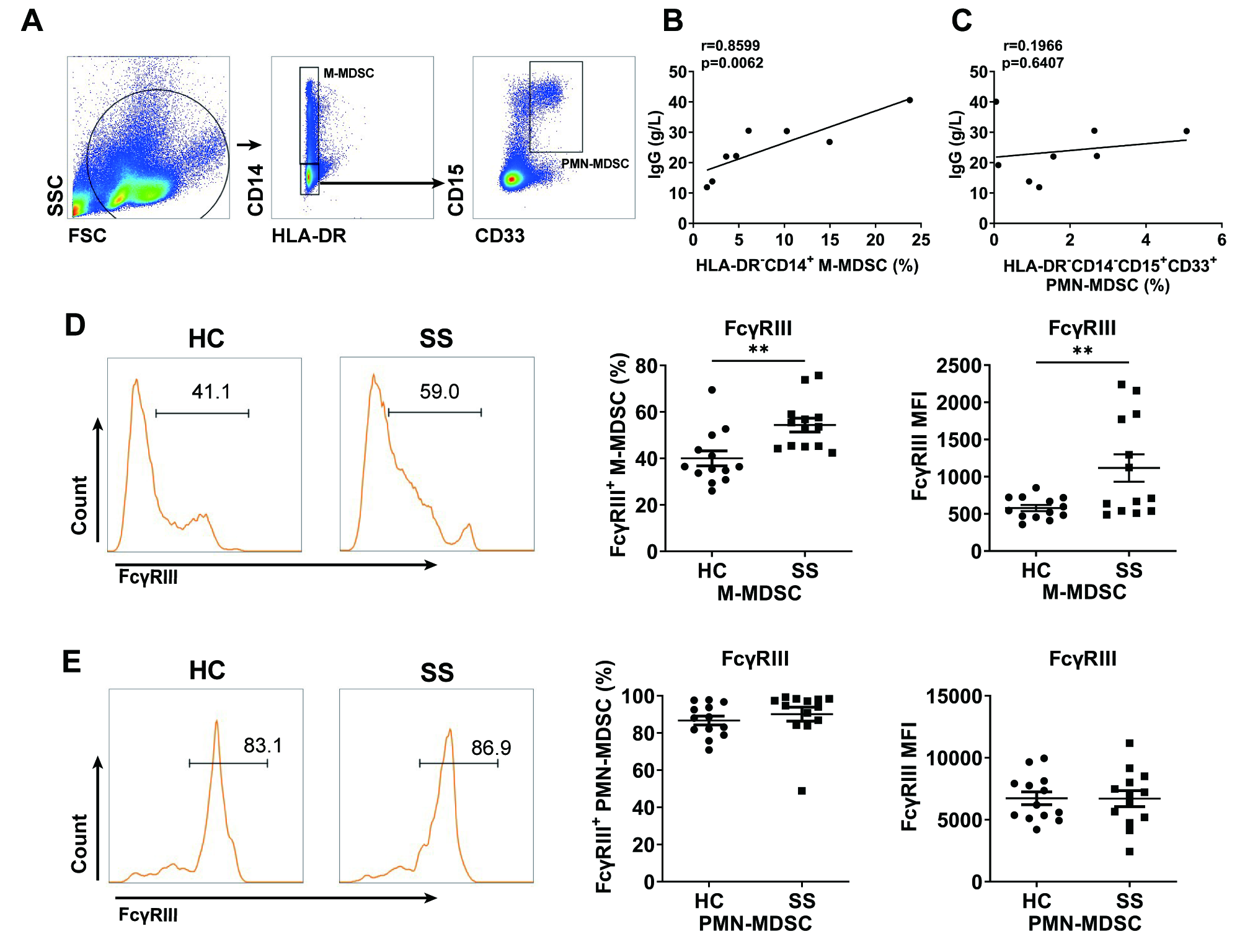


**Fig. S2. M-MDSCs** **expressed higher levels of FcγRIIIA** **in SS patients. (A)** The strategy of HLA-DR^-^CD14^+^ M-MDSCs and HLA-DR^-^CD14^-^CD33^+^CD15^+^ PMN-MDSCs gating. **(B-C)** The correlations of M-MDSCs and PMN-MDSCs with serum IgG were analyzed in SS patients (n=8). Representative flow cytometric analysis and percentages of FcγRIII^+^ M-MDSCs and the MFI of FcγRIII on M-MDSCs **(D)** and PMN-MDSCs **(E)** from HC and SS patients were shown (n=13). **p<0.01.

**
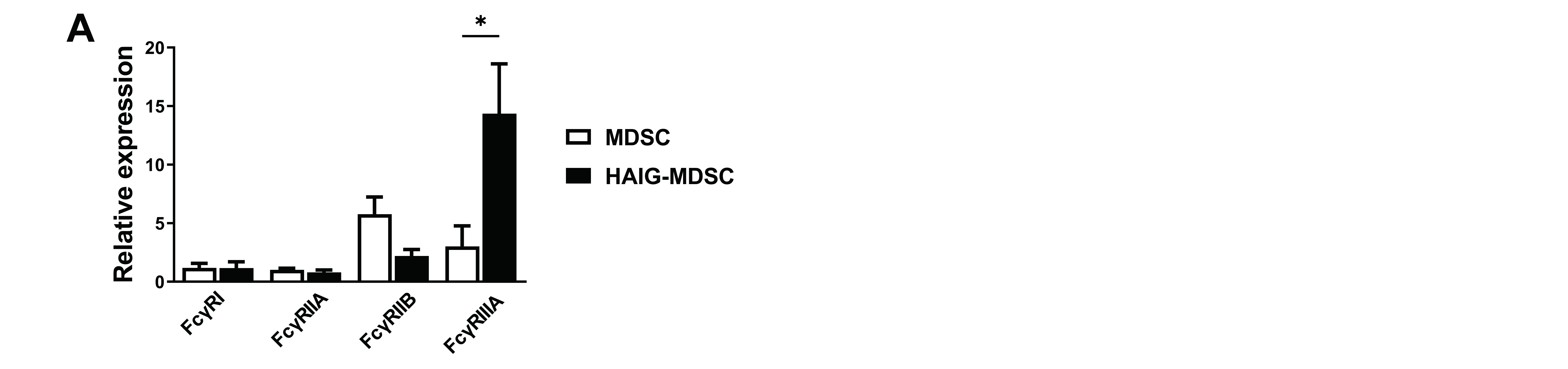
**

**Fig. S3.** **IgG-containing ICs increased the expression of FcγRIIIA in MDSCs.** MDSCs were stimulated with or without HAIG for 24 h. **(A)** The mRNA expressions of FcγRI, FcγRIIA, FcγRIIB and FcγRIIIA in MDSCs and HAIG-MDSCs were showed. All the data are representative of two independent experiments. n=4, *p<0.05.

**
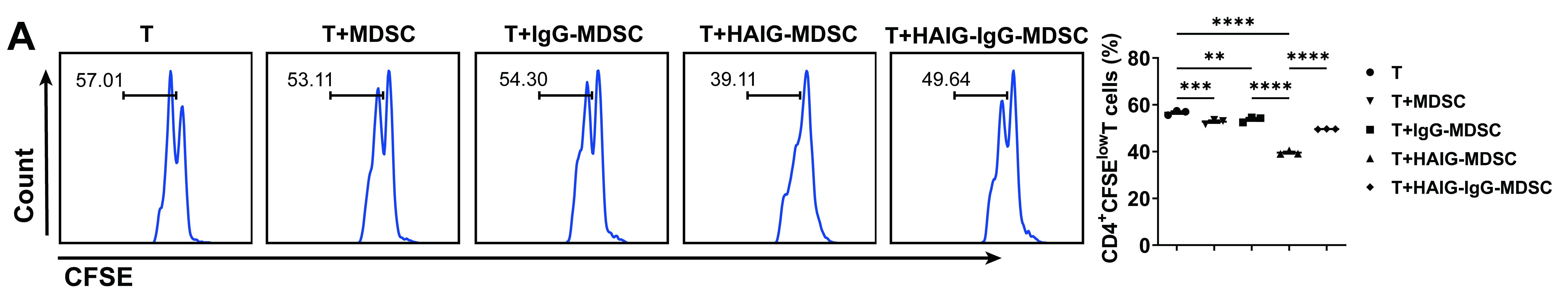
**

**Fig. S4. FcγRIIIA blockade decreased** **the suppressive ability of MDSCs in CD4^+^ T cell proliferation.** MDSCs or IgG-preprocessed MDSCs were stimulated with or without HAIG for 24 h, and then co-cultured with CFSE labeled CD4^+^ T cells for 72 h. **(A)** Representative flow cytometric analysis and percentages of CD4^+^CFSE^low^ T cells were showed. All the data are representative of two independent experiments. n=3, **p<0.01, ***p<0.001, ****p<0.0001.

**
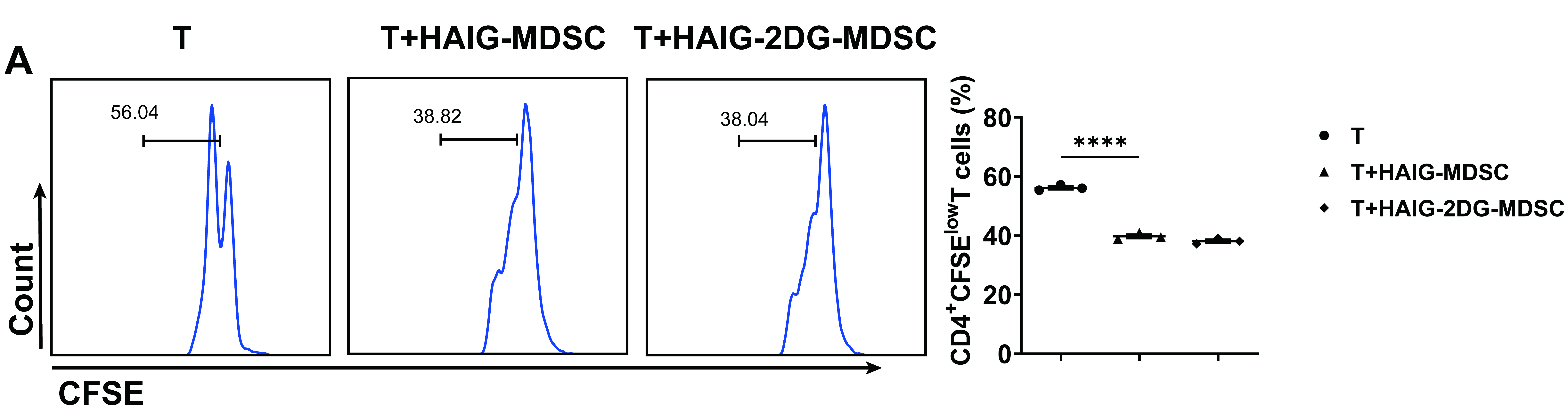
**

**Fig. S5. Glycolysis inhibition showed no effects on the suppressive ability of MDSCs on CD4^+^ T cells proliferation.** MDSCs or 2-DG-preprocessed MDSCs were stimulated with or without HAIG for 24 h, and then co-cultured with CFSE labeled CD4^+^ T cells for 72 h. **(A)**  Representative flow cytometric analysis and percentages of CD4^+^CFSE^low^ T cells were showed. All the data are representative of two independent experiments. n=3, ****p<0.0001.


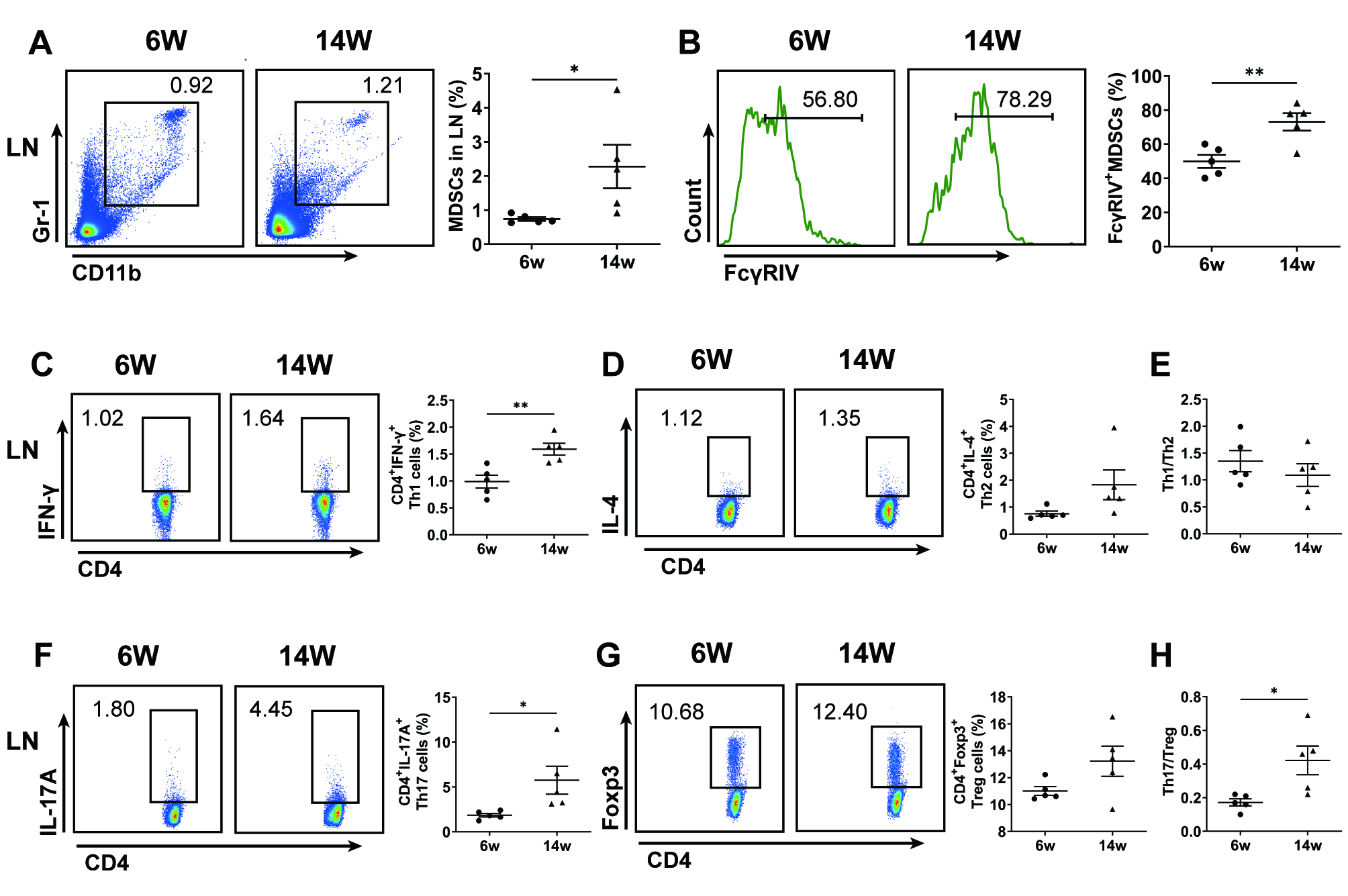


**Fig. S6. MDSCs from lymph node highly expressed FcγRIV in SS-like NOD mice.** CD11b^+^Gr-1^+^ MDSCs, CD4^+^IFN-γ^+^ Th1 cells, CD4^+^IL-4^+^ Th2 cells, CD4^+^IL-17A^+^ Th17 cells and CD4^+^Foxp3^+^ Treg cells in lymph node (LN) from 6 and 14-week-old NOD mice were detected by flow cytometry. Representative flow cytometric analysis and percentages of **(A)** MDSCs and **(B)** FcγRⅣ^+^ MDSCs were shown. Representative flow cytometric analysis and percentages of CD4^+^ T cell subsets were shown. **(C)** Th1 cells, **(D)** Th2 cells, **(E)** the ratio of Th1/Th2, **(F)** Th17 cells, **(G)** Treg cells and **(H)** the ratio of Th17/Treg were showed. All the data are representative of two independent experiments. n=5, *p<0.05, **p<0.01.

**Table S1 Clinical and laboratory characteristics of the SS patients.**

| Features | Values |
| --- | --- |
| Age (years) | 53.32 ± 2.04 |
| Disease duration (months) | 58.86 ± 6.70 |
| Female | 50 (100%) |
| ESSDAI | 5.66 ± 3.67 |
| IgG (g/ml) | 16.11 ± 0.52 |
| Anti-SSA antibodies | 32/50 (64%) |
| Anti-SSB antibodies | 19/50 (38%) |
| ANA | 31/50 (62%) |

**Table S2** **Primer sequences for real-time PCR.**

| Genes | Primers |
| --- | --- |
| GAPDH-F | 5’-CGAGATCCCTCCAAATCAA-3’ |
| GAPDH-R | 5’-TTCACACCCATGACGAACAT-3’ |
| GLUT1-F | 5’-CGGGCCAAGAGTGTGCTAAA-3’ |
| GLUT1-R | 5’-TGACGATACCGGAGCCAATG-3’ |
| HK2-F | 5’-TTGACCAGGAGATTGACATGGG-3’ |
| HK2-R | 5’-CAACCGCATCAGGACCTCA-3’ |
| LDHA-F | 5’-ATCTTGACCTACGTGGCTTGGA-3’ |
| LDHA-R | 5’-CCATACAGGCACACTGGAATCTC-3’ |
| Arg-1-F | 5’-GGCTGGTCTGCTTGAGAAAC-3’ |
| Arg-1-R | 5’-ATTGCCAAACTGTGGTCTCC-3’ |
| IL-1β-F | 5’-AAAGCTTGGT GATGTCTGGTC-3’ |
| IL-1β-R | 5’-GGACATGGAGAACACCACTTG-3’ |
| IL-6-F | 5’-CCTTCGGTCCAGTTGCCTTCTC-3’ |
| IL-6-R | 5’-CCAGTGCCTCTTTGCTGCTTTC-3’ |
| COX-2-F | 5’-TGACCAGAGCAGGCAGATGAA-3’ |
| COX-2-R | 5’-CCACAGCATCGATGTCACCATAG-3’ |
| IL-4-F | 5’-TTTGCTGCCTCCAAGAACACA-3’ |
| IL-4-R | 5’-TGTCGAGCCGTTTCAGGAAT-3’ |
| FcγRI-F | 5’-AATGGCACCTACCATTGCTC-3’ |
| FcγRI-R | 5’-TGTCACAGATGCATTCAGCA-3’ |
| FcγRIIA-F | 5’-GACTACGGATACCCAAATGTC-3’ |
| FcγRIIA-R | 5’-AAGCCAGCAGCAGCAAAA-3’ |
| FcγRIIB-F | 5’-TGACTGCTGTGCTCTGGGCG-3’ |
| FcγRIIB-R | 5’-AGCCTTTGGGGGAGCAGGTGT-3’ |
| FcγRIIIA-F | 5’-AAAAGCCACACTCAAAGATAGC-3’ |
| FcγRIIIA-R | 5’-GGTCCTTCCAGTCTCTTGTTGA-3’ |
